# Supplementary material for: Effect of mode of healthcare delivery on job satisfaction and intention to quit among nurses in Canada during the COVID-19 pandemic
Source: PLOS Glob Public Health. 2023 Dec 5;3(12):e0002675. doi: 10.1371/journal.pgph.0002675 (PMC10697541; doi:10.1371/journal.pgph.0002675)
Supplement: Attachment — Submitted filename: Research article review (PGPH).docx [file pgph.0002675.s001.docx]

**Research article review matrix**

**PGPH-D-23-01038**

**Title of research article:** ***Effects of mode of healthcare delivery on job satisfaction and intention to quit among Nurses in Canada.***

| **No** | **Initial comment after review** | **Suggestions** |
| --- | --- | --- |
| 1 | Improper arrangement of key words | Key words should be properly arranged in alphabetical order. Also, authors need to remove Canada as it is not a key word |
| 51 | Combined years (20202023) | Authors should consider using the appropriate year |
| 63-65 | Statement require at least two or more citations. | Authors need to consider citing relevant references to statement |
| 84 | “A study conducted in Turkey” | Authors need to identify the type of study and include in statement |
| 94 | Statement of “no study” had been conducted not strong enough to justify the study | Authors to provide equally important facts to justify the study |
| Table 2 | In the multivariate model (Regression model), It appears the OR and confidence interval values are higher than the values recorded for the adjusted odds ratio and corresponding CI for variables such as 10-19 years, multiple locations and others | Authors should critical look at the regression outcome and correct the report.  Ideally, once we don’t expect the unadjusted odds ration to be higher than the adjusted odds and the CI as well |
| 199 | Outpatients | Authors can consider separating them |
| 235 | Statement began with “Also” | Consider replacing with a “Again”, “Additionally” |
| **General impression** (Minor revision) | | Very insightful and informative paper as it highlights the need for healthcare organizations to address clinical nurses' job satisfaction and challenges, particularly during pandemics, to improve continuity of care. However, the authors need to address the concerns raised, particularly the regression results (output). |
