# Supplementary material for: Effect of mode of healthcare delivery on job satisfaction and intention to quit among nurses in Canada during the COVID-19 pandemic
Source: PLOS Glob Public Health. 2023 Dec 5;3(12):e0002675. doi: 10.1371/journal.pgph.0002675 (PMC10697541; doi:10.1371/journal.pgph.0002675)
Supplement: Attachment — Submitted filename: PGPH-D-23-01038 Response to reviewers.docx [file pgph.0002675.s003.docx]

PGPH-D-23-01038
Effect of mode of healthcare delivery on job satisfaction and intention to quit among nurses in Canada
PLOS Global Public Health

Response to Reviewers

| **Reviewer 1 comments** | **Authors response** |
| --- | --- |
| Very insightful and informative paper as it highlights the need for healthcare organizations to address clinical nurses' job satisfaction and challenges, particularly during pandemics, to improve continuity of care. However, the authors need to address the concerns raised, particularly the regression results (output) and the ones below: | Thank you for your positive assessment and your feedback for improving the paper. We updated the manuscript to address your concerns and described the changes below. |
| Line 1: Improper arrangement of key words - Key words should be properly arranged in alphabetical order. Also, authors need to remove Canada as it is not a key word | Keywords were re-arranged in alphabetical order and Canada was removed from the list. |
| Line 51: Combined years (20202023) - Authors should consider using the appropriate year | We apologize for this. The date was automatically changed when we converted and uploaded the file. We have fixed this. |
| Line 63-65: Statement require at least two or more citations - Authors need to consider citing relevant references to statement | Two references were added to support our statement. |
| Line 84: “A study conducted in Turkey” - Authors need to identify the type of study and include in statement | A sentence describing the study was added to the manuscript. |
| Line 94: Statement of “no study” had been conducted not strong enough to justify the study - Authors to provide equally important facts to justify the study | Arguments justifying the need for this study were added to the manuscript. |
| Table 2: In the multivariate model (Regression model), It appears the OR and confidence interval values are higher than the values recorded for the adjusted odds ratio and corresponding CI for variables such as 10-19 years, multiple locations and others - Authors should critical look at the regression outcome and correct the report. Ideally, once we don’t expect the unadjusted odds ration to be higher than the adjusted odds and the CI as well | Thank you for the note.  We double-checked all the unadjusted OR and adjusted OR values for every variable, and they are all correct.  Usually adjusted ORs are reduced when compared to the crude ORs, however, it is possible that crude ORs underestimate the true OR. Hence, when confounding variables are adjusted for in the models, the adjusted OR could be higher than the crude ORs. |
| Line 199: Outpatients - Authors can consider separating them | The reviewer raises an interesting point to assess the association between mode of healthcare delivery and intention to quit job separately for those who received training in infection prevention and those who did not.  The % of those who did not receive training was low at 8% and hence we would not have enough power to stratify by that variable. However, we believe that the results obtained, suggesting that those who did not receive training in infection prevention were at increased odds for quitting job is important, thus the justification for keeping this variable in the model. |
| Line 235: Statement began with “Also” - Consider replacing with a “Again”, “Additionally” | This word “Also” was replaced by “Additionally”. |
|  | Thank you for valuable feedback. |
|  |  |
| **Reviewer 2 comments** | **Authors response** |
| Thank you for the opportunity to review this manuscript. This manuscript describes the effect of mode of healthcare delivery on job satisfaction and intention to quit among nurses in Canada and reports nurses’ intention to quit within two years due to lack of job satisfaction. The mode of healthcare delivery was categorized into; in-person, online, or blended. I think this is a context specific and unique sample, and analyses on this sample have the potential to contribute to our understanding of in-person, online, or blended healthcare delivery and the effect it has on job satisfaction and the intention to quit during pandemics that has not been extensively studied for previous pandemics public good. My main concern is that there is no research question and or hypotheses presented. | Thank you for your positive assessment and your feedback for improving the paper. We updated the manuscript to address your concerns and described the changes below. |
| 1. The author should provide research question or hypotheses | The research question has been added to the introduction section of the revised manuscript. |
| 2. The author should correct the year in line 51 as there seem to be a typographical error | We apologize for this. The date was automatically changed when we converted and uploaded the file. We have fixed this. |
| 3. The author should provide intext citation for the statement in line 232 | Additional references in support of the statement were added to the manuscript. |
| 4. The article tittle may be modified to contain pandemic or COVID-19 to make it apt. This is because the mode of healthcare delivery and intention to quit was during the Covid-19 pandemic. | Thank you for your suggestion. We have modified the title of the paper. |
|  | Thank you for your valuable feedback. |
