# Supplementary material for: Effect of mode of healthcare delivery on job satisfaction and intention to quit among nurses in Canada during the COVID-19 pandemic
Source: PLOS Glob Public Health. 2023 Dec 5;3(12):e0002675. doi: 10.1371/journal.pgph.0002675 (PMC10697541; doi:10.1371/journal.pgph.0002675)
Supplement: Attachment — Submitted filename: REVIEWER COMMENT.docx [file pgph.0002675.s002.docx]

**SUMMARY OF THE RESEARCH AND YOUR OVERALL IMPRESSION**

Thank you for the opportunity to review this manuscript. This manuscript describes the effect of mode of healthcare delivery on job satisfaction and intention to quit among nurses in Canada and reports nurses’ intention to quit within two years due to lack of job satisfaction. The mode of healthcare delivery was categorized into; in-person, online, or blended. I think this is a context specific and unique sample, and analyses on this sample have the potential to contribute to our understanding of in-person, online, or blended healthcare delivery and the effect it has on job satisfaction and the intention to quit during pandemics that has not been extensively studied for previous pandemics public good. My main concern is that there is no research question and or hypotheses presented.

**RECOMMENDATION**

1. The author should provide research question or hypotheses
2. The author should correct the year in line 51 as there seem to be a typographical error
3. The author should provide intext citation for the statement in line 232
4. The article tittle may be modified to contain pandemic or COVID-19 to make it apt. This is because the mode of healthcare delivery and intention to quit was during the Covid-19 pandemic.

**IMPRESSION**

The study is worthy of acceptance and publication
