# Supplementary material for: Effect of mode of healthcare delivery on job satisfaction and intention to quit among nurses in Canada during the COVID-19 pandemic
Source: PLOS Glob Public Health. 2023 Dec 5;3(12):e0002675. doi: 10.1371/journal.pgph.0002675 (PMC10697541; doi:10.1371/journal.pgph.0002675)
Supplement: Attachment — Submitted filename: REVIEWER COMMENT 27102023-.docx [file pgph.0002675.s004.docx]

**SUMMARY OF THE RESEARCH AND YOUR OVERALL IMPRESSION**

Thank you for the opportunity to review this manuscript. This manuscript describes the effect of mode of healthcare delivery on job satisfaction and intention to quit among nurses in Canada during the COVID-19 pandemic and reports nurses’ intention to quit within two years due to lack of job satisfaction. The mode of healthcare delivery was categorized into; in-person, online, or blended. I think this is a context specific and unique sample, and analyses on this sample have the potential to contribute to our understanding of in-person, online, or blended healthcare delivery and the effect it has on job satisfaction and the intention to quit during pandemics that has not been extensively studied for previous pandemics for public good. Review recommendations have been addressed.

**IMPRESSION**

The study is worthy of acceptance and publication
